# Allowing for unemployment in productivity measurement

**DOI:** 10.1007/s43546-020-00008-7

**Published:** 2020-11-02

**Authors:** Rob Gandy, Chris Mulhearn

**Affiliations:** 1 Liverpool Business School, Liverpool John Moores University, Redmonds Building, Brownlow Hill, Liverpool, L3 5UG Merseyside UK; 2 13, Woodkind Hey, Wirral, CH63 9JY Merseyside UK

**Keywords:** Covid-19 pandemic, Economic index, Labour hoarding, Labour productivity, Social labour productivity index, Unemployment

## Abstract

The labour productivity index is a mainstay measure for comparing countries’ relative economic performance, but the Covid-19 pandemic could expose some of its inherent limitations: it focuses on people in work and ignores unemployment, and it is not standardised. In theory, a country’s index value could increase, even though its GDP might fall, because of significant increased unemployment in low-productivity sectors such as tourism and retail. It follows that the index value could fall when these sectors recover. Also, high-performing countries could see their index value fall because of the pandemic’s impact in high-value sectors, such as demand for oil.Consequently, a wider perspective of productivity is necessary. This paper, therefore, proposes a complementary index which adjusts labour productivity for levels of unemployment—the social labour productivity index (SLPI)—and recommends that the labour productivity index itself should be standardised. The relationship between employment and productivity is complex. For example, the UK’s economic performance, involving comparatively low labour productivity and low unemployment, has been deemed a ‘productivity puzzle’. A literature review discusses this relationship, but it is clear that econometric worldwide evaluation requires very large data sets, that are unlikely to be routinely available in practice to monitor international performance. By contrast, data sets on national productivity are small and already available, although they contain little or no data on causal factors. SLPI values were calculated for differing levels of unemployment and relative labour productivity for newly employed workers for countries where data was available; with patterns over the period 1986–2016 established for the G7 countries, Portugal, Ireland, Greece, and Spain. There were marked variations between the two indices for countries with high unemployment. The SLPI presents a practicable measure which can be utilised quickly in these unprecedented times. Using available data to compare countries’ GDP with their total workforce, it arguably provides a better measure of their overall economic and social health. Sensitivity analyses varying assumptions can model differing potential scenarios to sit alongside GDP and labour productivity index predictions.

## Introduction

The economic theory of productivity measurement goes back to the work of Jan Tinbergen (1942) and independently, to Robert Solow (1957), and is continually evolving [Organisation for Economic Co-operation and Development (OECD) 2001]. A wide range of productivity-related indicators are currently utilised [Office for National Statistics (ONS) 2016a]. One mainstay indicator for comparing countries’ relative economic performance is the labour productivity index. However, the Covid-19 pandemic could expose some of its inherent limitations: it focuses on people in work and ignores unemployment, and it is not standardised. The pandemic is expected to have far-reaching and long-term effects on the global economy with significant increases in unemployment, particularly in low-productivity service sectors such as tourism, leisure, and retail which employ lower-skilled workers (see below). In theory, this could mean that the rate of reduction in a country’s GDP is exceeded by its rate of increase in unemployment, with the possible consequent counter-intuitive effect that the value of the labour productivity index actually increases. A corollary is that the index value could fall if and when these low-productivity sectors recover. Also, there could be major volatility in some countries’ GDPs where the pandemic impacts on high-value sectors, such as the demand for oil (Barbosa et al. 2020), leading to high-performing countries seeing their index values fluctuate and/or fall as a result. Such volatility would serve to illustrate why the labour productivity index itself should be standardised. Given these circumstances and unprecedented times, a wider perspective of productivity is necessary. This paper, therefore, proposes a practicable complementary index which uses available data to compare countries’ GDP with their total workforce, thereby adjusting labour productivity for levels of unemployment. It is considered that this social labour productivity index (SLPI) provides a better measure of countries’ relative overall economic and social health.

## Impact of Covid-19 pandemic on world economy

At the time of writing (July 2020), the full impact of the Covid-19 pandemic on the world economy and individual countries is a matter for conjecture, with many projections being made with varying degrees of certainty. It is fair to say that the outlook is generally pessimistic. For example, the International Labour Organization initially reported that the impact could cause the equivalent of 195 million job losses (United Nations News 2020) due to the full or partial lockdown measures that had been put in place, which affected almost 2.7 billion workers, i.e., four in five of the world’s workforce. The four sectors likely to experience the most ‘drastic’ effects of the disease and falling production were: food and accommodation (144 million workers), retail and wholesale (482 million); business services and administration (157 million); and manufacturing (463 million). These sectors were described as being at the ‘sharp end’ of the impact of the pandemic, and account for 37.5% of global employment; and they all mainly involve high employment and low productivity. Yet within a very short time, the International Labour Organization (2020) increased its projected job losses to the equivalent to 305 million, due to the prolongation and extension of lockdown measures, with the situation worsening for all major regional groups. Particular concern was expressed that the worst-hit industries and services in low- and middle-income countries have high proportions of low-wage workers in informal employment, with limited access to health services and State welfare safety nets (United Nations News 2020). However, the pandemic impacted on the major G7 economies as well as the rest of the world, with jobless totals varying between countries; ranging from 1.76 million in Japan to 30 million in the United States (Kretchmer 2020).

Oil and natural gas extraction has long been the sector with the highest productivity, but this too has been severely hit by the pandemic because of reduced demand because of lockdowns; with air and car travel significantly reducing. This has led to the industry experiencing its third price collapse in 12 years. Following the first two shocks, the industry rebounded and business returned to usual, but the Covid-19 pandemic combined a supply shock with an unprecedented demand drop when the sector’s financial and structural health was worse than in previous crises. The advent of shale, excess supply, and generous financial markets that overlooked the limited capital discipline all contributed to poor returns. As a result, in May 2020, share prices touched 30-year lows (Barbosa et al. 2020). The knock-on effect on employment in some oil-producing centres has been marked; foreign workers in the Gulf were ‘stranded, unemployed and forgotten’, with reports of some being deported (Brennan 2020). Together with increasing societal pressure for renewable energy and reduced dependency on fossil fuels (Neill 2019), arguably change is inevitable and the pandemic crisis could be seen to be one of the industry’s most transformative moments, with the impact on productivity and employment uncertain (Barbosa et al. 2020).

Another high-productivity sector is real-estate activities, and property markets around the world will also probably suffer significantly due to the pandemic, because large unemployment, wage cuts, business failures, and job uncertainty will mean many people are likely to be cautious about investing in a home. This in turn can lead to falling house prices, as witnessed in the UK, USA, and many other countries during the last recession and credit crunch (Bloom 2020).

Should outbreaks of the pandemic persist, should restrictions on movement be extended or reintroduced, or should disruptions to economic activity be prolonged, the anticipated recession could be deeper than predicted. Businesses might have difficulty in servicing debt, heightened risk aversion could lead to climbing borrowing costs, and bankruptcies and defaults could lead to financial crises in many countries (World Bank 2020). Some countries’ economies have high reliance on sectors which are vulnerable to the impact of the pandemic. For example, tourism is particularly important for Spain and Italy, which accounts for 14.3% and 13.0%, respectively, of their GDP (McCarthy 2020), and this involves sectors that have been highlighted above as being the most adversely affected by the pandemic. Countries instigated measures to encourage (safe) tourism from mid-2020, but Christine Lagarde, the European Central Bank president, advised that ‘the economic recovery from the pandemic hit would be “restrained” as households save instead of spending’. She warned that some airlines and hotels would suffer ‘irredeemable’ damage (Business telegraph 2020). In respect of food, a wave of [United Kingdom (UK)] restaurant insolvencies was anticipated to be caused by the pandemic, following on from losses at the top 100 restaurant groups, which increased by 94% to £151 m in the previous year. Of those that survive, a number were likely to look at reducing the number of sites, cut menus, and make further redundancies, especially once the UK furlough scheme ended (Jones 2020).

By way of comparison, some manufacturers maintained their business (to some degree) by taking the opportunity to switch their production to commodities relevant to the pandemic, such as personal protective equipment. For example, over 100 UK manufacturers of all shapes and sizes responded positively to the government’s call for help building ventilators (Williamson 2020).

There can be no doubt that the impact of the pandemic across the world will be significant economically, and will probably have long-lasting consequences for some countries and some sectors. The above points are illustrative and cannot be totally exhaustive, particularly as there is uncertainty about whether there will be further waves (Centre for Evidence-Based Medicine 2020) and what might be the associated impact. Also, the capacity for governments to mitigate the worst effects of the pandemic was uncertain at the time of writing (Bank of England 2020; BBC News 2020; Her Majesty’s Government 2020; KPMG 2020). Nevertheless, there can be confidence that: productivity values in many sectors will change, some on a long-term basis; each country’s profile in terms of its balance of sectors will evolve; GDP values will fall (some temporarily and some more long-term); and unemployment will rise. It is therefore very likely that countries’ labour productivity indices will fluctuate more widely and frequently over a short period than in the past; with the counter-intuitive possibility that some will have their index value increase even though their GDP has reduced, if their unemployment rate has markedly increased enough, with low-productivity sectors bearing the brunt of job losses. Therefore, it is important that consideration can be given to how a country’s GDP relates to its whole (available) workforce, and not just those who are actually employed. This is why the SLPI is proposed.

## Literature review

Any literature review relating to productivity and unemployment cannot be totally exhaustive and it must be kept in mind that some research findings may not readily translate into the post-Covid-19 world. This literature review examines the UK’s ‘productivity puzzle’, discusses some employment practices that can affect productivity, and highlights some relevant international dynamics, such a migration. There are inter-relationships between these (and other) topics and there will be variations in how they affect different countries. However, it is important to bear in mind that immigration, whether it be legal or illegal, or involve high-skilled technical workers or low-skilled refugees, may well increase the size of the available workforce, but it will not necessarily increase the numbers in work (at least proportionately). It is the difference between those in work and the total workforce that the SLPI looks to address.

### UK’s ‘productivity puzzle’

**Fig. 1:**
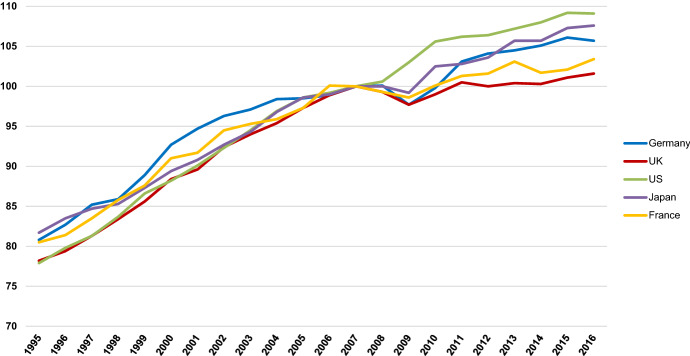
Productivity, constant price, GDP per hour worked, 2007 = 100 Source: Office for National Statistics (2018a)

There is unanimity in the literature that productivity matters: it is the key to rising living standards (Haldane 2017; Tenreyo 2018). There is also wide agreement that the UK’s labour productivity (Gross Domestic Product (GDP) per hour worked) is problematic both in absolute terms, since it stalled around the time of the 2008 financial crisis, and in relative terms, in that it is some way adrift of the rates achieved by the UK’s principal competitors (see Fig. 1). This has led to comments such as the following:

‘If British workers were able to catch-up to the G7 average, what currently takes us five days’ work to produce could be done in a little over four. If we were able to catch up with Germany, we might all be able to go home from work on Thursday afternoon each week without any fall in GDP.’ (Tenreyo 2018, p.5).

**Fig. 2:**
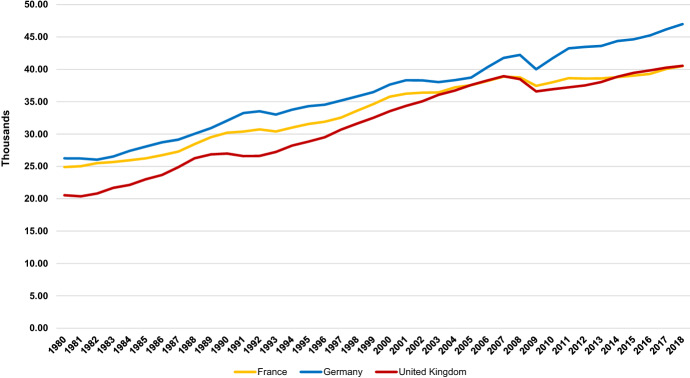
GDP per head, constant prices PPP 2011 int. dollar, 1980–2018 Source: International Monetary Fund (2019)

However, if the UK’s labour productivity is so adrift over the medium-to-long term, there should be evidence of its impact on living standards. GDP per head is the most common measure of living standards, and is one of the three components of human development—and arguably the most important—used by the United Nations (UN) in its annual calculations of the progress of the world’s economies (United Nations Development Programme 2018). Figure 2 compares British, French, and German GDP per head over almost 40 years. Two things stand out. First, the gap between the UK and France, which was evident in 1980, had closed by the time of the financial crisis in 2008 and, in 2018, the UK had the slightly higher figure. Second, although German GDP per head remained above that of the UK, the gap had not widened; indeed, at times it narrowed. Neither of these things should be evident in the presence of a secular decay of UK labour productivity.

In these circumstances, it is reasonable to infer that there may be conceptualization and measurement problems with GDP per head or with UK labour productivity. It is argued in this paper that a re-conceptualization of productivity that takes into account differences between countries’ unemployment levels is a step towards providing a more nuanced interpretation of how productive economies actually are. It is important to note that issues in the labour market are but one possible contribution to an understanding of the UK’s ‘productivity puzzle’. Other interrelated dimensions of what is almost certainly a multiply-determined phenomenon include: problems of measurement that tend towards an underestimation of UK labour productivity (Bean 2016); crisis-related scarring that has ossified resource allocation (Haldane 2017); insufficient work-related training (Dearden et al. 2006); and low private and public investment (Kierzenkowski et al. 2018) where private investment may be inhibited by distortions imposed by the corporate tax system (Bournakis and Mallick 2018a, b).

### Labour hoarding

One theoretical influence on the value of a country’s labour productivity rests on the concept of labour hoarding. Labour hoarding is the retention of workers by firms during periods of slow economic growth or outright recession during which the expected dominant process might be labour shedding (Bryson and Forth 2016; Crawford et al. 2013**)**; the likely impact being to dampen both productivity and unemployment rates. The founding theorization of this process is due to work in the 1960s by Arthur Okun (Biddle 2014) who argued that, in contrast to the long-held classical view, labour was not a factor of production that could be fired and rehired without cost. Dismissal and recruitment necessitate resource commitments: these processes are not free. Neither are they perfect substitutes: firms’ employees develop skills and aptitudes that may be permanently lost should redundancies occur in an economic downturn. The rationale for labour hoarding becomes even stronger in an environment where real wages are depressed and the direct cost of retaining workers falls.

Labour hoarding may be part of the explanation of the labour productivity slowdown experienced by several European economies and the UK in particular since the 2008 financial crisis and the recession that followed (Dolphin and Hatfield 2015). Evidence from the UK shows that, in the aftermath of the crisis, firms tended to retain workers, but constrain wages and investment; this was particularly true of smaller firms that invested in training (Crawford et al. 2013; MAKEuk 2018). At the same time, firms that did shed labour tended to release less-skilled workers whilst retaining those with higher skills (Martin and Rowthorn 2012). Despite the depth of the recession in 2008–09 (a contraction of some 4.6%), and the extraordinary length of time it took the economy to regain its previous peak (6 years), UK unemployment increased less than it had done in the comparatively milder recessions in the early ‘80 s and early ‘90 s. This was arguably because (many) firms chose to hoard labour, thereby to some extent limiting their capacity to squeeze productivity increases from the labour process: hence the link between labour hoarding and flat UK labour productivity. The inverse happened during, for example, the ‘Thatcher’ recession of the early 1980s. Then, a 2.8% contraction of the economy resulted in a peak unemployment rate of almost 12%; but this dramatic labour shedding was associated with the sharpest short-term jump in UK labour productivity in the last 50 years.

A complementary way to conceptualise the relationship between labour hoarding and productivity is to accept that the UK’s low productivity is a reflection of its preference for retaining low-productivity and low-paid jobs. This, in turn, would account for the stagnation of UK real wages since the financial crisis. Not all sectors may be thus characterised as there is evidence of productivity dispersion between UK firms, but the long tail of low-productivity-labour-hoarding firms may account for the secular trend (Faggio et al. 2010).

Moreover, if national differences in labour hoarding and shedding provide a partial explanation of the productivity puzzle, possibly we should be less worried about productivity gaps between economies because, as it stands, ‘pure’ productivity is an incomplete conceptualization of a complex phenomenon. For example, as noted, after 2008 UK firms retained people in work in the teeth of an exceptional downturn. Why? Following Okun’s argument (Biddle 2014): because firms supposed it competitive so to do. Observe also the wider emollient effect this had on the economy as the abrasions and social aggravations associated with high and prolonged unemployment were limited. To take a contrasting case, in France, productivity and unemployment were higher than in the UK both in the aftermath of the 2008 financial crisis and across recent decades. In productivity terms, the French performance may be enviable but the implications of higher unemployment—such as Okun’s costs of labour turnover, the problems of hysteresis and wider social costs—sit, as it were, outside the equation and are ignored. As will be seen in “Results”, what the SLPI shows is that, when adjusted for unemployment, French productivity is necessarily attenuated, because the SLPI offers the beginnings of an approximation of such costs. It also suggests that countries that retain labour (and labour hoarding might be only one reason to do so) are not as adrift in economic terms as pure productivity comparisons suggest.

### Hysteresis effects of unemployment

It is important to take account of unemployment when considering labour productivity, because high unemployment and underemployment have high economic and social costs—through so-called hysteresis effects—to drain vitality from economic recovery (Blanchard and Summers 1986; Cross 1993, 2000; O’Shaughnessy 2011). Short-term job losses can become long-term unemployment as unemployment-generating structural changes in labour markets—and therefore in the economy as a whole—become path-dependent (Ball 2009; Røed 1997). The most durable explanation of this process arises from the struggle between insiders (the employed) and outsiders (the unemployed and those on the margins of work) in the labour market (Blinder 1988). The market power of insiders in a depressed labour market allows them to retain their jobs as it is they who largely determine wage-setting (and not the outsiders who are effectively disenfranchised) (Blanchard and Summers 1986). The chief source of the insiders’ market power stems from the labour turnover costs that firms would incur in trying to dispense with the services of skilled and experienced workers (Lindbeck and Snower 1986). One implication of hysteresis is its enervating impact on outsiders. The long-term unemployed lose skills and the habits of work and may be cut adrift from the productive economy. More widely, hysteresis may produce an economy trapped in stagnant growth and apparently permanently high unemployment, both of which undermine social cohesion (International Labour Organization—International Monetary Fund 2010).

### Investment in education

Education is a prime driver of productivity and there are risks of government expenditure levels on education falling in the Covid-19 pandemic crisis. Nevertheless, the rates of return to education may rise, as with other crises, since earnings for the less-educated fall because of increased unemployment rates amongst this group which in turn suppresses their wages. If university graduates’ earnings remain unchanged, or only change marginally, then the rate of return to university education can increase during crises; particularly as better-educated workers can more easily find other work to maintain earnings. Less-educated workers are likely to take lower-paying jobs during crises, whilst more advantaged graduates may switch more quickly to better jobs. Also, employers may be more likely to retain educated workers, because they are more adaptable to changing economic conditions (Fasih et al. 2020).

### Youth unemployment

Linked to the education-related issues is that of youth unemployment, which has been an ongoing problem in countries across the world, and is likely to be exacerbated by the Covid-19 pandemic. This was already a problem in mid-2014 when the youth unemployment rate in the eurozone reached 24% (up from 15% in 2007) compared to 10.25% for adults (up from 6.50% in 2007) (Banerji et al. 2015 p. 6). Youths are three times more likely than adults to be unemployed, and over 350 million young people are not engaged in education, employment, or training. Research has shown that the highest unemployment rate in 25 of 27 developed countries was amongst people with primary education or less, and the fact remains that those who have a lower level of education are less likely to be employed. The demographic bulge of about 3.5 billion global citizens below the age of 25 (Hannah 2014) means that youth unemployment should not be ignored in relation to a country’s productivity.

### Migration and productivity

There have been substantial immigration flows into several European countries, such as the United Kingdom, Germany, Spain, and Italy, with the evidence pointing to small wage effects and possibly some negative employment effects on nationals. However, there are substantial variations between European countries both in the presence of immigrants and their labour market policies and institutions, and will affect the impact and absorption of immigrants. One noted phenomenon, especially in Germany and the UK, is an apparent ‘skill downgrading’ of immigrants, i.e., better-educated workers performing jobs comparable to less-skilled nationals. This might be due to barriers created by language, licensing, and legal requirements; but it suggests that immigrants competing more with less-educated nationals than similarly educated nationals (Peri 2016). Yet, it is equally important to look at the impact of emigration on economies. For example, Greece has been hampered in its economic recovery from its first bailout programme in 2010, because of the ‘brain drain’ of thousands of its educated citizens moving abroad and then not necessarily returning (Hope 2018). Also, the emigration of skilled workers has lowered firms’ productivity performance in Eastern European countries (Giesing and Laurentsyeva 2017).

In addition, it should be noted that migrants can fall into quite distinct groups, as reflected by US immigrants being overrepresented at the two extremes of the skill distribution (the less-educated and better-educated); with underrepresentation at intermediate levels. In 2014, one-third of US workers with a PhD and a job in a STEM (science, technology, engineering, and mathematics) field were foreign-born, with a possible factor being that mathematics–analytical skills are more easily transferred across countries than managerial and communication skills, which are more culture and country specific. In comparison, immigrants represented 40% of workers with no high school diploma. Such immigration of the better-educated may improve learning and promote innovation at the local level with positive productivity effects. It should also be noted that foreign students are the fastest-growing group of foreign-born, and tertiary education is likely to be a sector of significant growth for jobs, value-added, and (service) exports for the US economy. Foreign students increase the demand for such services and, once students graduate with a US degree, they can be well positioned to be productive workers and professionals (Peri 2016).

### Refugees and productivity

A distinction is being made between the general migration issues described above and those of refugees. This is because whilst the latter are also migrants their situations and needs are quite different. At the end of 2019, there were nearly 26 million refugees worldwide (from amongst 79.5 million who were ‘forcibly displaced’), with around half under the age of 18. More than half of refugees were from four countries: Syria (6.6 million), Venezuela (3.7 million), Afghanistan (2.7 million), and South Sudan (2.2 million), and the major host countries for refugees were: Turkey (3.6 million), Colombia (1.8 million), Pakistan (1.4 million), Uganda (1.4 million), and Germany (1.1 million) (United Nations Refugee Agency 2020).

The large number of refugees and migrants has tested the European Union’s (EU) cohesion and decision-making ability. However, given Europe’s ageing and shrinking population, migrants are seen by some to be necessary to replace missing nationals; thereby maintaining its capacity to keep its economies strong and its welfare systems sustainable (Martín et al. 2016). There are also macroeconomic benefits to activating refugees which are not only budgetary. It has been estimated that there will be a positive medium-term impact of the related increased labour supply. Taking reasonable assumptions, it was projected that the aggregate impact on EU GDP could be as high as 0.25 percentage points by 2020; and for the main destination countries such as Germany, Sweden, and Austria, it could be as high as 0.5–1.1 additional percentage points. This would be huge for EU growth standards: equivalent to, for instance, the estimated impact of EU Structural Funds in the growth rate in Spain in the 1990s. However, this was dependent upon the assumptions about the labour market integration of refugees (Martín et al. 2016).

Notwithstanding this perceived opportunity, Europe has struggled to cope with a large-scale influx of migrants with divisions within the EU over how best to support refugees. The crisis had a disproportionate impact on some countries (the largest intakes were to Germany, Italy, and France) and there were calls for more collaboration at EU level to coordinate their integration (Persaud 2017). However, the demographic profiles of refugees are skewed towards the young and men: men constituted 60% of asylum applicants from the ten largest countries of origin, with the figure rising to over 90% for Bangladesh and Pakistan; 83% of the first time asylum seekers were younger than 35 years old, with those aged 18–34 years accounting for 51% and 32% being minors younger than 18 years. Unfortunately, refugees struggle to locate employment commensurate with their skills and, consequently, the process of integration is often associated with downward professional mobility (Robila 2018).

Different countries set up different integration programmes for immigrant economic integration and this has had a big impact on outcomes. Some Scandinavian countries developed extensive state-sponsored integration programmes, which included housing and employment assistance. But even countries with long experience of integrating asylum seekers (Sweden and Denmark) have found that less than one-third of refugees are employed after 3 years (Martín et al. 2016). By comparison, for example, the UK sent asylum seekers to excluded urban areas which have an excess of available housing but not necessarily job opportunities. Cognisance should also be taken of where economic incentives to access the labour market may be distorted by the benefits to which refugees (and asylum seekers) are entitled; this should be taken into account in the design of any potential labour market integration support measure (Martín et al. 2016).

It has been found that migrants entering their country of asylum through international protection and asylum processes have employment rates lower on average compared than migrants in general. This is mainly due to their qualification and skills profile, but the impact of the psychological trauma many have suffered also plays a part (Martín et al. 2016). Even so, where asylum seekers and refugees have both skills and qualifications they can experience high levels of unemployment (where they were allowed to work) and those employed work in low-skilled jobs with earnings below the average (Robila 2018).

### Illegal immigration

A precondition for the existence of illegal labour immigration is the existence of an informal economy. This includes where there are non-declared activities, which have a lower-than-normal price, being agreed by the buyer and seller knowing non-declaration shares savings in taxes and duties, and similar. The extent to which this affects a country will vary; some countries such as Portugal have marked numbers of illegal workers (mostly from former colonies and because of weak trade unions), whereas Scandinavian countries have been able to contain this problem (Hjarnø 2019). Yet even where laws have been put in place with increased penalties, such as the UK, the non-legal labour market continues to grow. To avoid exposure to the authorities (and possible deportation), illegal immigrants work in the shadow economy; they are paid in cash, are subject to low wages and poor conditions, and have no benefits or rights. Many illegal immigrants are brought to the country by employers, tying the workers to a life of inhumane conditions and no prospects (Security Watchdog 2017). Some illegal immigrants will actually be the victims of human trafficking for sex, organ harvesting, and modern-day slavery, which has taken the lives of many adults and children over the years (Cotom 2020). All such labour is by definition not regulated and, therefore, difficult to measure, but the estimated cost to the UK treasury is £2 billion a year, or 7% of GDP (Security Watchdog 2017). Data on numbers of illegal immigrants in individual countries will not be part of official employment statistics, and will always be the subject of research and estimation.

## Social labour productivity index

### Allowing for unemployment when measuring productivity

The ONS (2018b) invited bids for small research funding grants to encourage research in the development of statistics to better reflect the economic environment as it evolves in multifaceted ways making economic measurement ever more complex. This was because of the often digitally delivered growth in services, the importance of intangible assets, and the continuing trend of globalisation, all making the understanding and quantifying of economic activity more challenging. One of the stated areas of interest for these grants was:

‘improving the quality and scope of productivity statistics to deliver a world-class set of statistics to support users attempting to address the “productivity puzzle”’

Now, labour productivity is an internationally utilised economic index which measures how efficiently labour input is combined with other factors of production and used in the production process. Labour input is defined as total hours worked of all persons engaged in production. Labour productivity only partially reflects the productivity of labour in terms of the personal capacities of workers or the intensity of their effort. The ratio between the output measure and the labour input depends to a large degree on the presence and/or use of other inputs (e.g., capital, intermediate inputs, technical, organisational and efficiency change, and economies of scale).

The research covered by this paper emanated from the fact that labour productivity only relates to those people in work, i.e., it excludes the unemployed. Therefore, there are likely to be differential impacts on countries’ productivity figures according to the degree to which the various factors referred to above apply. Also, people with high skills will be numbered amongst the unemployed when they are between jobs, but many (if not most) people who are unemployed in any country will be people with relatively low-skill profiles who are only likely to obtain jobs that have relatively low productivity.

The multiple factors that can affect the relationship between productivity and unemployment, as outlined, are manifold and complex; and past economic relationships and processes may change considerably as a result of the Covid-19 pandemic. Some historical research and data may not readily translate for future projections. Ideally, there will be much econometric research to re-evaluate and re-determine the inter-relationships between the various factors and how to economically measure productivity in such a new environment. Unfortunately, this will involve a great deal of research effort and resource, and probably take several years. Given these circumstances, the proposed SLPI can act as a straightforward proxy measure that can be readily utilised. Its values can be calculated for differing (assumed/projected) levels of unemployment and levels of labour productivity for newly employed workers across those countries for which appropriate data are publically available. The SLPI can then act as a useful complementary index to labour productivity, which can be further developed (see Further Research below).

### Balancing unemployment and productivity

One issue that the SLPI seeks to address is the fact that some countries with comparatively high labour productivity internationally can also have comparatively high unemployment rates, as in the quoted case of the UK in the early 1980s. Should countries that have high(er) productivity at the expense of high(er) unemployment be applauded compared to those that appear productive ‘laggards’ but manage to achieve, as the UK did prior to the Covid-19 pandemic, a situation as close to full employment as anything seen since the end of the post-WWII boom? It is important to take account of unemployment when considering labour productivity, because high unemployment and underemployment can have high economic and social costs, if unaddressed because of potential hysteresis effects (Blanchard and Summers 1986; Cross 1993, 2000; O’Shaughnessy 2011). Therefore, by allowing for different levels of unemployment when measuring countries’ productivity levels, the SLPI may provide a better yardstick of their economic and social fitness.

The SLPI might also shed some light on the apparent inconsistency between Figs. 1 and 2. Paul Krugman’s well-known aphorism that *in the long-run productivity is nearly everything* implies that because of the UK’s inferiority in Fig. 1, its performance in Fig. 2 is hard to understand (Krugman 1994). Widening our conceptualization of productivity is a step towards overcoming this problem. Productivity adjusted for unemployment allows us to begin to account for an array of hidden costs lurking outside the traditional productivity data but which impacts, potentially heavily, on economic performance. Figure 1 neglects these costs, thus flattering the prowess of countries—like France—which are more burdened by them. The UK’s inconsistency across the two figures can now be explained. The UK may be behind in pure productivity terms, but its more recent medium-term retention of labour suggests that it escapes a series of economic costs its competitors incur, meaning that its material prosperity is higher than might be inferred from its place in the international productivity hierarchy.

### The relationship between productivity and unemployment

A country’s overall economic and social health cannot be measured solely by its labour productivity. Measuring social health is complex and includes employability, marital satisfaction, sociability, and community involvement, and links to psychological well-being and other psychological measures. Socially healthy people may, because of their social health, more likely enjoy good physical health and psychological well-being, compared to people who are dissatisfied with their marriages, socially isolated, and/or relatively unemployable (Renne 1974). That health underpins wealth is reflected in the notion that healthier nations are wealthier nations (Contoyannis and Forster 1999), with there being two-way relationships between economic growth and health: economic growth acts as stimulus for good health and good health is considered a key determinant of growth (Deaton 2000). Also, improvements in health and education both lead to increased worker productivity (Ullah et al. 2019). However, that there is a positive association between unemployment and a range of measures of ill health has long been known, although the relationship between the two is not straightforward (Griffin 1993; Norström et al. 2019; Watkins 1992). With the predicted increases in unemployment worldwide due to the Covid-19 pandemic (see above), there is a risk that this will have a deleterious impact on people’s health with knock-on effects on productivity, which in turn could then lead to further unemployment; a potential vicious circle. In the circumstances, it makes sense to monitor productivity in a way that accounts for unemployment to gain a wider, more rounded perspective. This would require access to regularly published national data, but much of that necessary to appropriately accommodate all (or even a selection) of the factors mentioned above is not necessarily collected. Ideally, the way forward would involve a considerable amount of standardised data and an econometric approach. Unfortunately, the requisite large data sets are simply not available in practice, e.g., for reasons of confidentiality/competitiveness in the private sector. Furthermore, there would be much debate about exactly what would be the most appropriate data, given that most insights have been gained by individual research, which have focused on particular disciplines, issues, and research questions, often in a single country. The development and agreement of such data and how it should be analysed, on a worldwide basis, would take a long time. Therefore, to make early progress, any initial step should look to utilise existing data, as far as possible, whilst taking account of the fact that data sets on national productivity are small and contain little or no data on causal factors.

**Fig. 3:**
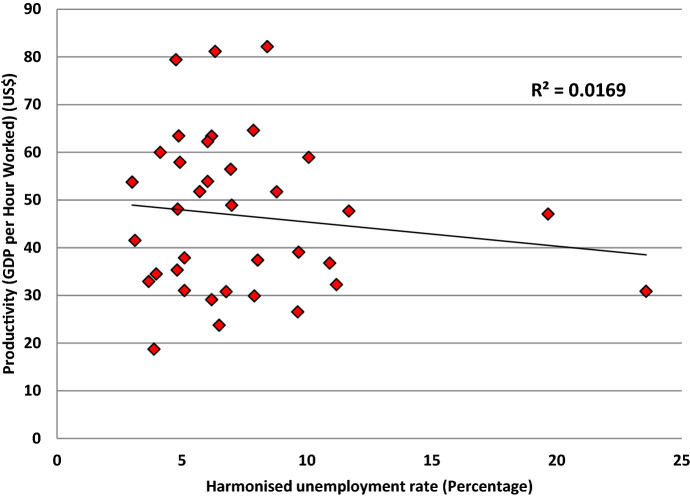
Relationship between countries’ productivity and unemployment (2016)

One premise underpinning the proposed SLPI is the fact that some countries with ‘good’ labour productivity can have ‘poor’ unemployment rates. Therefore, comparing GDP with a country’s total workforce is arguably a better measure of a country’s overall economic and social health. Figure 3 shows the relationship between labour productivity, i.e., GDP per hour worked (GDPHRWKD) (in USA dollars), and the harmonised percentage unemployment rate (HUR) for the 36 countries for which such data were available for 2016 (OECD 2018a; b). What it demonstrates is that there is no correlation between the two indicators, with a correlation coefficient value of *R*^2^ = 0.016.

It is seen that the country with the highest productivity ($82.15) was Ireland, which had an HUR of 8.4%. The country with the lowest productivity ($18.74) was Mexico, which had an HUR of 3.9%. They are two very different countries, with very different profiles of economic activity, but they serve to illustrate the variations between countries and how productivity and unemployment are not directly related. One factor is that countries that have comparatively low unemployment will have found/created employment for those people who would otherwise have not obtained employment because of their relatively poor skills and qualifications. Therefore, the jobs involved will not necessarily be highly paid, and hence, this will drag down the overall labour productivity. The corollary is that countries with high labour productivity and high unemployment have the former artificially inflated because of the latter. In light of the above, the authors believe that the proposed SLPI presents an initial (proxy) means of examining and monitoring patterns of productivity and unemployment worldwide, which can be readily applied, because it uses data that is already collected and published. These patterns, and any associated dynamics over time, should enable governments and agencies to identify outliers and raise questions about the relationship which will help target future investigation, research, and actions.

### Assumptions for SLPI formula

As stated above, the purpose of the SLPI is to compare countries’ GDP with their total workforce, rather than just people in work, as does the labour productivity index. Therefore, it needs to take into account levels of unemployment whilst utilising readily available data. Table 1 sets out the basic formulaic assumptions utilised in creating the SLPI and the following section shows how it was developed. But first, an outline of the developmental approach is required. Each country will have its existing performance-related data, and each is given the suffix ‘e’ (for ‘existing’): *G*_e_ = published GDP; *P*_e_ = published labour productivity index; *T*_e_ = total existing full-time and part-time workers; *U*_e_ = existing number of unemployed; and *β*_e_% = existing unemployment rate. What the SLPI does is to make a projection of what productivity would be if there was a uniform (minimum) level of unemployment across all countries. Therefore, there needs to be ‘projected’ data and how these are calculated is shown in Table 1. All such data are given the suffix ‘p’: *G*_p_ = projected GDP; *P*_p_ = projected labour productivity index; *T*_p_ = total projected full-time and part-time workers; *U*_p_ = projected number of unemployed; *β*_p_% = projected unemployment rate; and NE_p_ = projected newly employed. However, it must be emphasised that the ‘projection’ relates to a projected alternative contemporary scenario rather than a projection into the future.

**Table 1:** Key formulaic assumptions

*A* = *T*_e_ + *U*_e_ = *T*_p_ + *U*_p_ *U*_p_ = *U*_e_ − NE_p_ NE_p_ = *U*_*e*_ – *U*_p_ = *T*_p_ − *T*_e_ β_e_% = Unemployment Rate_e_ = (*U*_e_ × 100)/*A* β_p_% = Unemployment Rate_p_ = (*U*_p_ × 100)/*A* Employment Rate_e_ = *T*_e_/*A* Employment Rate_p_ = *T*_p_/*A* *U*_p_ = (*β*_p_% × *A*)/100 Productivity_e_ = GDP_e_/*T*_e_ Productivity_p_ = GDP_p_/(*T*_e_ + NE_p_) GDP_p_ = GDP_e_ + GDP of newly employed workers GDP of newly employed workers = (α × *P*_e_) × NE_p_ GDP_p_ = GDP_e_ + ((α × *P*_e_) × NE_p_) = GDP_e_ × (*T*_e_ + (α × NE_p_))/*T*_e_	

*A* all available workers, *G* GDP, *NE* newly employed workers, *P* productivity, *T* total full-time and part-time workers, *U* no. unemployed

What is critical is that a factor α is needed. This reflects the reasonable assumption that if a country with high labour productivity and high unemployment were to create work that helps to (significantly) reduce unemployment, then on average the pay and productivity of the new jobs, and therefore the newly employed, will be lower than those already in existence. Consequently, α will have a value < 1, to reflect the specific labour productivity of the newly employed relative to the existing labour productivity. Therefore, the SLPI formula described below takes the ‘existing’ (published) values for the various related statistics, and then makes a ‘projection’ of what they would become if the stated assumptions were applied, for the same point in time. Of course, as countries’ GDP values vary from year to year, corresponding values of the SLPI would be calculated for each year’s GDP values.

The following key assumptions are made:

- A country’s existing GDP (GDP_e_) is fixed for the purposes of calculations (for any given point in time).
- The existing unemployment rate of *β*_e_% changes to an assigned/predicted (standard) unemployment rate of *β*_p_%
- The mean total hours worked of all persons engaged in production (THW) of existing workers and newly employed workers is the same (otherwise, a new factor γ would be required to distinguish the relative working hours patterns between new and existing workers; γ could be greater or less than 1); alternatively, it could be envisioned that this is covered by the value of α

### Development of SLPI formula

Set out below is the construction of the SLPI formula based on the above assumptions. The authors did not have access to the ideal, primary data and, therefore, necessarily utilised relevant publically available data. What is demonstrated is that the SLPI uses publically available data and is consistent with existing indices:

$${\text{SLPI}}\, = \,G_{{\text{p}}} /(T_{{\text{e}}} \, + \,{\text{NE}}_{{\text{p}}} ).$$

$$\, = \,\left( {G_{{\text{e}}} \, + \,\left( {\left( {\alpha \, x \, P_{{\text{e}}} } \right) \, x{\text{ NE}}_{{\text{p}}} } \right)} \right)/ \, \left( {T_{{\text{e}}} \, + \,{\text{NE}}_{{\text{p}}} } \right) \quad \left[P_{{\text{e}}} \, = \,G_{{\text{e}}} /T_{{\text{e}}} ;{\text{ and}}\;T_{{\text{p}}} \, = \,T_{{\text{e}}} \, + \,{\text{NE}}_{{\text{p}}}\right] ;$$

$$\, = \,\left( {\left( {\left( {G_{{\text{e}}} x \, T_{{\text{e}}} } \right)\, + \,\left( {\alpha \, x \, G_{{\text{e}}} x \, NE_{{\text{p}}} } \right)} \right)/ \, T_{{\text{e}}} } \right)/ \, T_{{\text{p}}} .$$

$$\, = \,\left( {G_{{\text{e}}} x \, \left( {T_{{\text{e}}} \, + \,\left( {\alpha \, x \, NE_{{\text{p}}} } \right)} \right)} \right)/ \, (T_{{\text{e}}} x \, T_{{\text{p}}} ).$$

$$\, = \,P_{{\text{e}}} x \, \left( {T_{{\text{e}}} \, + \,\left( {\alpha \, x{\text{ NE}}_{{\text{p}}} } \right)} \right)/ \, T_{{\text{p}}} \quad \left[ {\text{NE}}_{{\text{p}}} \, = \,T_{{\text{p}}} - T_{{\text{e}}}\right] .$$

$$\, = \,P_{{\text{e}}} x \, \left( {\left( {T_{{\text{e}}} \, + \,\left( {\alpha \, x \, \left( {T_{{\text{p}}} - T_{{\text{e}}} } \right)} \right)} \right)/ \, T_{{\text{p}}} } \right) \quad \left[ {\text{Multiply numerator and denominator by}}\;A \right] .$$

$$\, = \,P_{{\text{e}}} x \, \left( {\left( {T_{{\text{e}}} \, + \,\left( {\alpha \, x \, \left( {T_{{\text{p}}} - T_{{\text{e}}} } \right)} \right)} \right)/ \, T_{{\text{p}}} } \right) \, x \, (A/A).$$

$$\, = \,P_{{\text{e}}} x \, \left( {A/T_{{\text{p}}} } \right) \, x \, (\left( {T_{{\text{e}}} /A} \right)\, + \,\left( { \, \alpha \, T_{{\text{p}}} /A} \right) - \left( { \, \alpha \, T_{{\text{e}}} /A} \right)).$$

$$\, = \,P_{{\text{e}}} x \, \left( {A/T_{{\text{p}}} } \right) \, x \, (\left( {\left( {\left( {1 - \, \alpha } \right)xT_{{\text{e}}} } \right)/A} \right)\, + \,\left( {\alpha \, T_{{\text{p}}} /A} \right)).$$

$$\, = \,P_{{\text{e}}} x \, \left( {\left( {\left( {1 - \, \alpha } \right)x{\text{ Employment Rate}}_{{\text{e}}} } \right)\, + \,\left( { \, \alpha \, x{\text{ Employment Rate}}_{{\text{p}}} } \right)} \right)/{\text{Employment Rate}}_{{\text{p}}} .$$

$${\text{Because Employment Rate}}_{{\text{e}}} \, = \,\left( {1 - \beta_{{\text{e}}} } \right){\text{ and Employment Rate}}_{{\text{p}}} \, = \,(1 - \beta_{{\text{p}}} ).$$

$${\text{SLPI = Existing Labour Productivity }} \times \frac{{\left( {\left( {1 - {\mkern 1mu} \alpha } \right)\left( {1 - {\mkern 1mu} \beta _{{\text{e}}} } \right)} \right){\mkern 1mu} + {\mkern 1mu} \left( {\alpha {\mkern 1mu} \left( {1 - {\mkern 1mu} \beta _{{\text{p}}} } \right)} \right){\text{ }}}}{{\left( {1 - {\mkern 1mu} \beta _{{\text{p}}} } \right)}}.$$

This formula enables the SLPI to be calculated for all countries using readily published data. It requires a minimum unemployment rate to be set, so that all countries are brought to an equal level, to establish the impact of the index. It represents the most basic version of the index, as it is important to first determine its veracity and potential to contribute to the debate about productivity before seeking greater sophistication; suggestions for the latter are presented in ‘Further Research’ below.

## Methodology

The methodology applied is primarily descriptive and empirical. This is because it was necessary to (a) determine whether the SLPI could actually be calculated, and (b) establish if the SLPI shows different patterns to the labour productivity index. The latter was important, because if the SLPI did not show any difference from the labour productivity index, then it would not be providing additional insight. As mentioned above an econometric approach which sought to establish causal relationships would be ideal, but unfortunately the limited availability of related international standardised data meant that this was outside the scope of this research. Nevertheless, it is anticipated that the usage of the SLPI could raise questions which would help target future econometric research.

The SLPI model was calculated for 2016 for countries across the world where data from the OECD were available for each of the GDPHRWKD (OECD 2018a) and the HUR (OECD 2018b). Values of α were varied for the full range of decile values from 0.1 to 0.9, and *β*_p_ took on values 3.0, 3.5, 4.0, 4.5, and 5.0, given that the lowest HUR was 3.01 (Iceland).

To examine patterns in the SLPI over time, it was decided to focus attention on historical data for some ‘sentinel’ countries. Those chosen were the G7 (Canada, France, Germany, Italy, Japan, the UK, and the United States of America) and the ‘PIGS’ countries (Portugal, Ireland, Greece, and Spain). The latter was chosen as a recognised counterbalance to the former, given their negative growth rates due to their banking sectors being severely hit by the 2008 financial crisis (OECD 2016). The additional years chosen were: 1986, 1991, 1996, 2001, 2006, and 2011 (the latter two to show the impact of the 2008 financial crisis). It was deemed appropriate to exclude East European countries because of their being communist states historically, and during part of the period covered by the analyses. (However, some consideration is afforded to East Germany, to the extent that it was absorbed by West Germany in the reunification process from 1989.)

It should be highlighted that standard values were set for *β*_p_ for the purposes of this paper to establish the comparative impact of standard values across all countries. However, it is fair to say that most countries with high levels of unemployment in 2016 [e.g., Greece (23.6%) and Spain (19.65%)] would struggle to attain such low levels. The next stage of development for the SLPI could include values for *β*_p_ being set for each country individually (see Further Research below). It was considered to be inappropriate to have undertaken such variations in values here, because it would simply have involved guesswork on the part of the authors. The analyses undertaken effectively represent sensitivity analyses of the SLPI, albeit within the stated limited range for *β*_p_. If non-standardised values of *β*_p_ were required for individual countries, then either they could be agreed as set target levels of unemployment, or a process of delphi methods could be applied to determine best predictions; given that they are well-established and have been used successfully for many purposes (Gupta and Clarke 1996; Linstone and Turoff 1975).

## Results

Table 2 shows the SLPI calculations for the 36 countries that had both the GDPHRWKD and the HUR for 2016. (Countries excluded because of insufficient data included Bulgaria, Croatia, Romania and Russia.) It only shows the SLPI calculations for α values of 0.5 and 0.1 (i.e., the mid-range and extreme values). This is for presentational purposes, because the differences in the SLPI calculations for each incremental 0.1 change in α values were constant for each country (but obviously varied between countries). The value for *β*_p_ shown was 3.0. The table shows the percentage reduction on the GDPHRWKD for each SLPI calculation to gauge the impact of using the SLPI rather than the GDPHRWKD. Finally, it shows the ranking of the 36 countries for each of the six figures. The countries are shown ranked for the percentage reduction achieved by the SLPI where α was 0.5. It is clear that the countries with the highest HUR see the SLPI values which have the greatest difference to the GDPHRWKD, which in part is the nature of the construction of the SLPI.

**Table 2:** Labour productivity, harmonised unemployment rate, and social labour productivity index (2016) by country

*β*_p_ = 3.0	GDPHRWKD	Harmonised unemployment rate (HUR)	SLPI (α = 0.5)	SLPI (α = 0.1)	% Reduction in GDPHRWKD to:		Rank					
					SLPI (α = 0.5) (%)	SLPI (α = 0.1) (%)	GDPHRWKD	Harmonised unemployment rate (HUR)	SLPI (α = 0.5)	SLPI (α = 0.1)	% Reduction in GDPHRWKD to:	
											SLPI (α = 0.5)	SLPI (α = 0.1)
Greece	30.87	23.57	27.60	24.98	**10.60**	19.09	30	1	33	33	**1**	1
Spain	47.08	19.65	43.04	39.81	**8.58**	15.45	19	2	19	20	**2**	2
Italy	47.71	11.68	45.57	43.86	**4.47**	8.05	18	3	18	18	**3**	3
Portugal	32.28	11.18	30.91	29.83	**4.22**	7.59	28	4	28	29	**4**	4
Turkey	36.79	10.91	35.29	34.09	**4.08**	7.34	24	5	24	26	**5**	5
France	58.95	10.07	56.80	55.08	**3.64**	6.56	9	6	10	10	**6**	6
Slovak Republic	39.06	9.67	37.72	36.64	**3.44**	6.19	21	7	21	22	**7**	7
Latvia	26.57	9.63	25.66	24.94	**3.42**	6.15	34	8	34	34	**8**	8
Finland	51.75	8.79	50.21	48.97	**2.98**	5.37	15	9	15	15	**9**	9
Ireland	82.15	8.41	79.86	78.03	**2.79**	5.02	1	10	1	3	**10**	10
Slovenia	37.41	8.03	36.44	35.66	**2.59**	4.67	23	11	23	23	**11**	11
Lithuania	29.91	7.9	29.15	28.55	**2.53**	4.55	32	12	31	31	**12**	12
Belgium	64.62	7.86	63.00	61.71	**2.51**	4.51	4	13	4	5	**13**	13
Canada	48.92	6.99	47.91	47.11	**2.06**	3.70	16	14	16	17	**14**	14
Sweden	56.45	6.95	55.30	54.38	**2.04**	3.66	11	15	11	11	**15**	15
Estonia	30.81	6.77	30.21	29.73	**1.94**	3.50	31	16	30	30	**16**	16
Chile	23.75	6.49	23.33	22.98	**1.80**	3.24	35	17	35	35	**17**	17
Luxembourg	81.16	6.33	79.77	78.65	**1.72**	3.09	2	18	2	1	**18**	18
Poland	29.12	6.19	28.64	28.26	**1.64**	2.96	33	20	32	32	**19**	19
Denmark	63.41	6.19	62.37	61.53	**1.64**	2.96	6	19	6	6	**20**	20
Austria	53.92	6.03	53.08	52.40	**1.56**	2.81	12	21	13	13	**21**	22
Netherlands	62.26	6.03	61.29	60.51	**1.56**	2.81	7	22	7	7	**22**	21
Australia	51.79	5.71	51.07	50.49	**1.40**	2.51	14	23	14	14	**23**	23
Hungary	31.03	5.1	30.70	30.43	**1.08**	1.95	29	24	29	28	**24**	25
New Zealand	37.89	5.1	37.48	37.15	**1.08**	1.95	22	25	22	21	**25**	24
Switzerland	57.93	4.92	57.35	56.90	**0.99**	1.78	10	26	9	9	**26**	26
United States	63.45	4.87	62.84	62.35	**0.96**	1.74	5	27	5	4	**27**	27
United Kingdom	48.12	4.83	47.66	47.30	**0.94**	1.70	17	28	17	16	**28**	28
Israel	35.33	4.81	35.00	34.73	**0.93**	1.68	25	29	25	24	**29**	29
Norway	79.42	4.76	78.70	78.12	**0.91**	1.63	3	30	3	2	**30**	30
Germany	60.01	4.13	59.66	59.38	**0.58**	1.05	8	31	8	8	**31**	31
Czech Republic	34.50	3.97	34.33	34.19	**0.50**	0.90	26	32	26	25	**32**	32
Mexico	18.74	3.88	18.66	18.59	**0.45**	0.82	36	33	36	36	**33**	33
Korea	32.93	3.67	32.82	32.73	**0.35**	0.62	27	34	27	27	**34**	34
Japan	41.54	3.12	41.51	41.49	**0.06**	0.11	20	35	20	19	**35**	35
Iceland	53.76	3.01	53.76	53.76	**0.01**	0.01	13	36	12	12	**36**	36
Euro area (19 countries)	53.00	10.03	51.08	49.54	**3.62**	6.52						
European Union (28 countries)	47.71	8.55	46.34	45.25	**2.86**	5.15						
OECD—total	47.06	6.34	46.25	45.60	**1.72**	3.10						

The countries are ranked in order according to their value of “Percentage Reduction in GDPHRWKD to SLPI (*α* = 0.5)” and therefore the columns with their calculations and ranking are highlighted for ease of reference

**Fig. 4:**
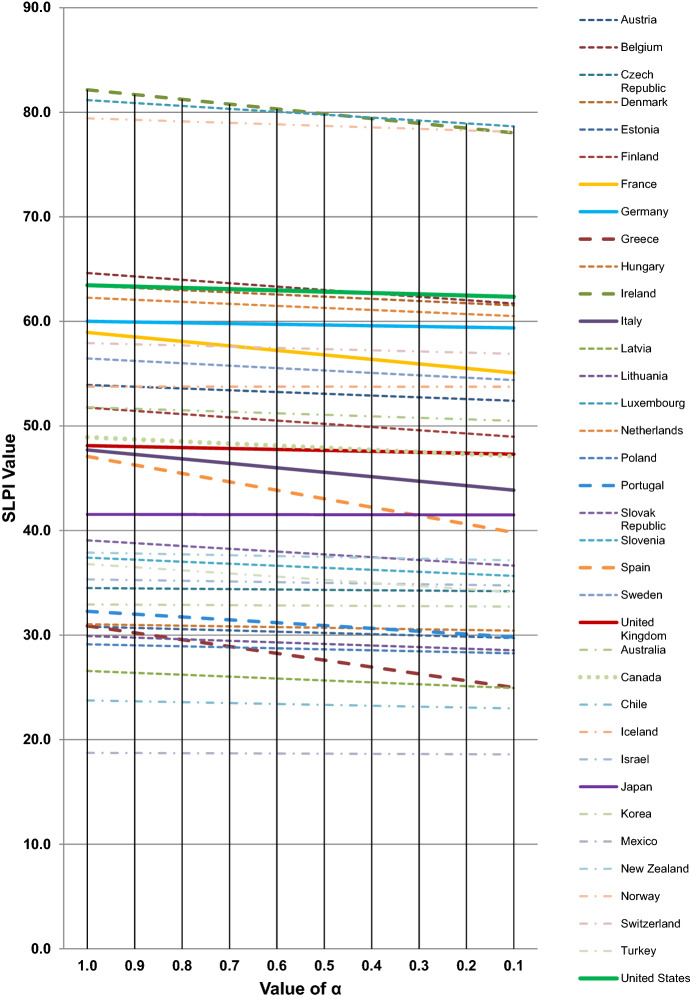
Calculated SLPI for assumed values of α (with *β*_p_ = 3.0) for all countries for which all data were available for 2016

Table 2 and Fig. 4, which show the SLPI values for each country for each incremental value of α, show great divergence between the different countries for both the GDPHRWKD [18.74 (Mexico) to 82.15 (Ireland)] and the HUR [3.01 (Iceland) to 23.6 (Greece)]. The SLPI values effectively are straight lines for each country, as would be expected. The GDPHRWKD range is so large that the calculation of the SLPI does not lead to any major variations in countries’ rankings. The three stand-out highest performers were Ireland, Luxembourg and Norway, which each were 23% or more greater than the fourth-ranked country (Belgium) for the GDPHRWKD and 25% or greater for the SLPI where α = 0.5. There were then different clusters of countries with broadly similar figures, with the lowest encompassing Mexico, Chile, and Latvia. However, it is noticeable that Greece moved from a higher group into this latter group for the SLPI because of its high unemployment, which at 23.57% meant that the SLPI (α = 0.5) was 10.60% lower than its GDPHRWKD.

The eight countries which had their SLPI (α = 0.5) value 3.00% or lower than their GDPHRWKD included two countries from the G7 (France and Italy) and three countries that were amongst the PIGS group, viz., Greece, Portugal, and Spain. Of course, for the many countries with low HUR figures, the SLPI showed little difference to the GDPHRWKD.

The research involved calculation of the SLPI for the following *β*_p_ values: 3.0, 3.5, 4.0, 4.5, and 5.0. It was found that, for SLPI (α = 0.5), the minimum value for *β*_p_ = 3.0 was 18.66 (Mexico) and the maximum was 79.86 (Ireland); for SLPI (α = 0.1), the minimum value for *β*_p_ = 3.0 was 18.59 (Mexico) and the maximum was 78.65 (Luxembourg). However, when *β*_p_ was increased to 5.0, the SLPI (α = 0.5) had a minimum value of 18.85 (Mexico) and maximum of 80.67 (Ireland); and SLPI (α = 0.1) minimum value was 18.94 (Mexico) and the maximum was 80.14 (Luxembourg). That the differences between corresponding SLPI figures for the different values of *β*_p_ were limited led to the decision to only present those in relation to *β*_p_ = 3.0 in the tables and figures.

However, it should be noted that when the value of *β*_p_ was increased, those countries that they had an HUR value less than *β*_p_ had SLPI values greater than the GDPHRWKD.

**Fig. 5:**
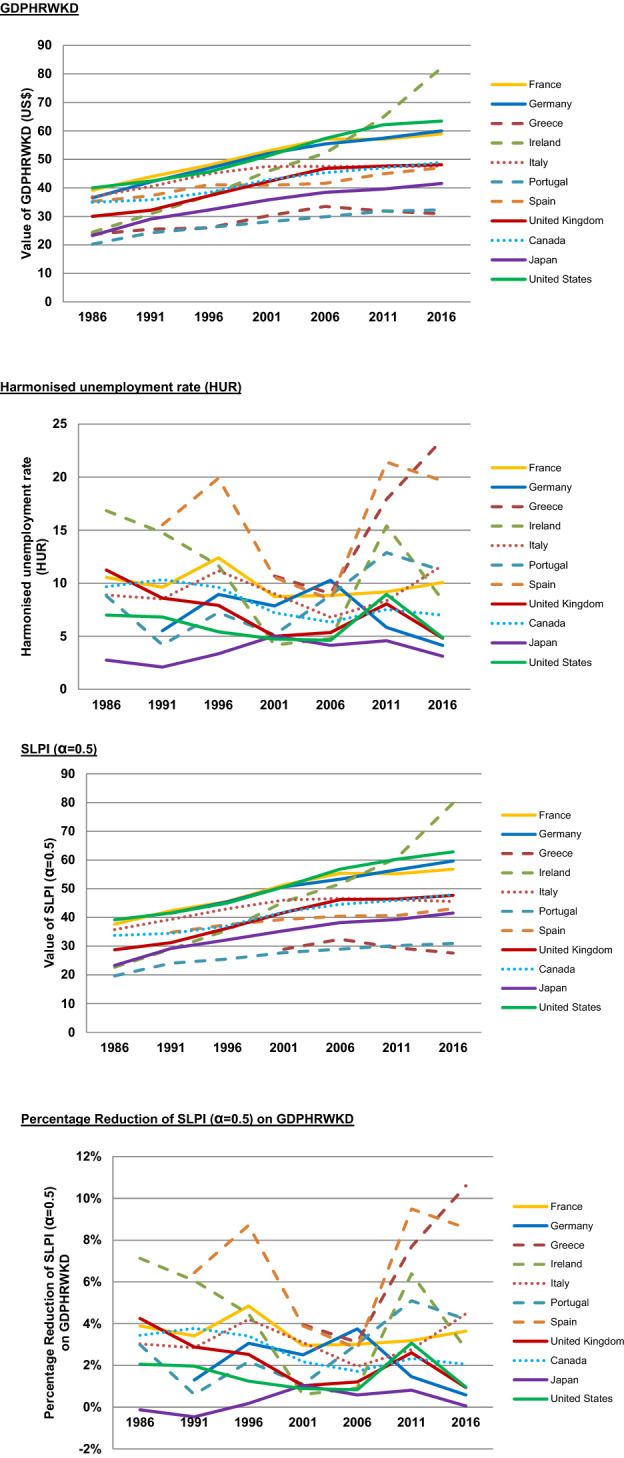
Trends over time (1986–2016) for G7 and PIGS countries for key indicators (with *β*_p_ = 3.0)

Table 3 provides details of the GDPHRWKD, the HUR, the SLPI (α = 0.5), and the percentage reduction of SLPI (α = 0.5) on the GDPHRWKD for the 11 chosen countries for each of 1986, 1991, 1996, 2001, 2006, 2011, and 2016. Figure 5 shows that GDPHRWKD and SLPI (α = 0.5) follow similar trajectories to one another. The HUR and the percentage reduction of SLPI (α = 0.5) also follow similar trajectories to one another, but they are markedly different from the other pair. This demonstrates that the SLPI (α = 0.5) is something of a refinement of the GDPHRWKD which responds to and reflects levels of unemployment (over time).

**Table 3:** Trends over time (1986–2016) for G7 and PIGS countries for key indicators (with *β*_p_ = 3.0)

GDPHRWKD	1986	1991	1996	2001	2006	2011	2016
France	39.2	43.9	47.9	52.9	57.1	57.0	58.9
Germany	36.5	42.1	46.9	52.0	55.4	57.4	60.0
Greece	23.7	25.5	26.0	30.1	33.5	31.9	30.9
Ireland	24.4	30.9	37.3	45.7	52.2	64.9	82.1
Italy	36.9	40.5	44.9	47.5	47.5	47.4	47.7
Portugal	20.3	24.2	26.1	28.1	29.9	31.8	32.3
Spain	35.2	37.2	41.1	40.9	41.6	44.9	47.1
United Kingdom	30.0	32.1	37.2	42.1	46.8	47.6	48.1
Canada	34.9	35.8	38.4	42.8	45.3	47.0	48.9
Japan	23.3	29.0	32.3	35.7	38.4	39.6	41.5
United States	40.0	42.4	45.6	51.1	57.3	62.1	63.5

Harmonised unemployment rate (HUR)	1986	1991	1996	2001	2006	2011	2016
France	10.6	9.6	12.4	8.7	8.8	9.2	10.1
Germany		5.5	8.9	7.9	10.3	5.8	4.1
Greece				10.7	9.0	17.9	23.6
Ireland	16.8	14.8	11.7	4.2	4.8	15.4	8.4
Italy	8.9	8.5	11.2	9.0	6.8	8.4	11.7
Portugal	8.8	4.2	7.2	5.1	8.9	12.9	11.2
Spain		15.5	19.9	10.6	8.5	21.4	19.7
United Kingdom	11.2	8.6	7.9	5.0	5.4	8.1	4.8
Canada	9.7	10.3	9.6	7.2	6.3	7.5	7.0
Japan	2.8	2.1	3.4	5.0	4.1	4.6	3.1
United States	7.0	6.8	5.4	4.7	4.6	9.0	4.9

SLPI (α = 0.5)	1986	1991	1996	2001	2006	2011	2016
France	37.66	42.36	45.59	51.32	55.39	55.21	56.80
Germany		41.51	45.45	50.65	53.33	56.58	59.65
Greece				28.95	32.43	29.44	27.60
Ireland	22.69	28.98	35.61	45.45	51.69	60.71	79.86
Italy	35.77	39.31	43.02	46.04	46.60	46.12	45.57
Portugal	19.66	24.07	25.51	27.76	28.97	30.20	30.92
Spain		34.81	37.52	39.29	40.43	40.68	43.04
United Kingdom	28.73	31.22	36.26	41.68	46.24	46.40	47.66
Canada	33.74	34.44	37.06	41.88	44.55	45.91	47.91
Japan	23.29	29.18	32.19	35.34	38.20	39.26	41.51
United States	39.18	41.54	45.00	50.67	56.86	60.23	62.84

Percentage reduction of SLPI (α = 0.5) on GDPHRWKD	1986	1991	1996	2001	2006	2011	2016
France	3.9%	3.4%	4.8%	3.0%	3.0%	3.2%	3.6%
Germany		1.3%	3.1%	2.5%	3.8%	1.5%	0.6%
Greece				4.0%	3.1%	7.7%	10.6%
Ireland	7.1%	6.1%	4.5%	0.6%	0.9%	6.4%	2.8%
Italy	3.0%	2.8%	4.2%	3.1%	2.0%	2.8%	4.5%
Portugal	3.0%	0.6%	2.2%	1.1%	3.0%	5.1%	4.2%
Spain		6.4%	8.7%	3.9%	2.8%	9.5%	8.6%
United Kingdom	4.2%	2.9%	2.5%	1.0%	1.2%	2.6%	0.9%
Canada	3.4%	3.8%	3.4%	2.2%	1.7%	2.3%	2.1%
Japan	− 0.1%	− 0.5%	0.2%	1.0%	0.6%	0.8%	0.1%
United States	2.1%	2.0%	1.2%	0.9%	0.8%	3.1%	1.0%

A sensitivity analysis was undertaken to establish the range of SLPI values that resulted from making the most extreme assumptions, within the ranges stated, for the 11 countries. This was between SLPI values for *β*_p_ = 5.0 and α = 0.9 and *β*_p_ = 3.0 and α = 0.1. The percentage difference of the latter from the former was (in descending order): Greece (17.5%); Spain (14.1%); Italy (7.4%); Portugal (7.0%); France (6.1%); Ireland (4.7%); Canada (3.5%); USA (1.7%); UK (1.7%); Germany (1.1%); and Japan (0.3%).

## Discussion

### Results

The results show that the SLPI is very relevant for countries that have high unemployment and that it reflects variations in unemployment levels over time. It does not make a great deal of difference from the GDPHRWKD for low unemployment countries, but, of course, its whole purpose is to suitably relate to countries’ whole population available for work. The observed wide distribution of GDPHRWKD draws the inference that there should be individual values of α for each country. This is because, in the short-term future at least, new jobs which take large numbers of people from unemployment are likely to be of a comparatively low productivity, particularly if they are in the service sector (Feldstein 2017). That unemployment will continue to be a factor in many countries following the Covid-19 pandemic, even some that had favourably low rates beforehand, is a reasonable assumption; particularly as chronic problems such as high youth unemployment were not fully successfully addressed in prior years.

The ratio of the service sector’s productivity against the prevailing overall productivity for each country will vary, and would be very noticeable for high performers such as Ireland, Luxembourg, and Norway. There would probably be lower values of α for countries with high GDPHRWKD and higher values for those with low GDPHRWKD.

There are potential options for determining individual values of α for each country. One is for there to be one (internationally) agreed sector that all such values relate to, e.g., the ‘Food And Beverage Service Activities’ sector; the value of α for each country would then be that country’s GDPHRWKD for ‘Food And Beverage Service Activities’ divided by its overall GDPHRWKD (which would allow for the fact that the GDPHRWKD for ‘Food And Beverage Service Activities’ will vary between countries). Another option would be for each country to designate which sector would be most likely to expand and take on its unemployed—which could vary between different countries. This would mean that the value of α for each country would then be that country’s GDPHRWKD for its ‘selected sector’ divided by its overall GDPHRWKD. Of course, for both such options, and any others, it might be preferable to use a combination of sectors rather than just one alone. This whole issue would be a subject for further research.

That the ranking of countries for the SLPI is virtually the same as the GDPHRWKD does not reflect a problem in the SLPI, but more of a problem with the GDPHRWKD. It is arguable that labour productivity in the ‘high performance’ countries is skewed: Ireland hosts the information technology headquarters of many multinational companies and has a significant inflow of foreign direct investment (the latter also applies to Luxembourg) (OECD 2016) and Norway’s economy relies heavily on the extraction of crude petroleum and natural gas, which is generally the highest labour productivity sector of all. Also, all three have comparatively small populations. Therefore, it could be argued that the GDPHRWKD values for these countries provide ‘false’ comparisons with larger countries, whose economies have greater emphasis on sectors which have lower productivity. Such extreme values arguably undermine the efficacy of the GDPHRWKD, which should be subject to some agreed form of standardisation (see below).

It can be seen that the SLPI can accommodate varying values of *β*_p_, even for countries where the HUR is lower. For example, Iceland, which had the lowest HUR value of 3.01, and had an SLPI (α = 0.5) value of 54.32 and an SLPI (α = 0.1) value of 54.77 compared to a GDPHRWKD of 53.76 when *β*_p_  = 5.0. This is not an issue, as it demonstrates that the SLPI implicitly assumes that (a) unemployment had increased and (b) the jobs shed were those of a lower productivity than the average for the country (the relationship determined by the value of α), meaning that the average increased accordingly. The sensitivity analysis comparing SLPI values for *β*_p_ = 5.0 and α = 0.9 and *β*_p_ = 3.0 and α = 0.1 served to highlight that there can be marked differences depending upon the assumptions made, particularly for countries with existing high unemployment. This in turn suggests that the values of α and *β*_p_ should be set for each country individually (see Further Research).

With regards to the various issues relating to unemployment and production (e.g., labour hoarding), the SLPI does not measure them per se; and some of them are qualitative issues rather than quantitative issues. To isolate each from the other would be very difficult, and so the SLPI is a high-level proxy measure aimed at countries’ whole workforces and economies.

### Need for standardisation of labour productivity

It is self-evident that labour productivity will vary between different economic sectors. The UK uses the Standard Industrial Classification SIC2007 (ONS 2019a; b) which is compatible with the European Community’s statistical classification of economic activities (NACE Rev.2) (European Commission 2019). Scrutiny of UK output per hour values (seasonally adjusted, at current prices and in £) for the 67 different industrial/ economic categories quoted in Quarter 4 of 2018 (ONS 2019b) showed very wide variations. These ranged from the minimum of £9.51 (Security and Investigation Activities) to the maximum of £1254.23 (Extraction of Crude Petroleum and Natural Gas). The median value was £35.00 (Computer Programming, Consultancy and Related Activities). However, it is notable that values for ‘Extraction of Crude Petroleum and Natural Gas’ can vary wildly—the Quarter 3 2018 value was £720.33! Unsurprisingly, this sector usually has the highest productivity of all UK sectors—only in three Quarters over the period 1997–2018 was its output per hour exceeded by another sector (‘Real Estate’ in each case).

The UK has been well known for being reliant on the service sector, and this is a marked change historically. The ONS (2016b) highlighted five facts about the UK service sector: 79% of UK GDP came from the service sector in 2013; the percentage of workers in the service sector rose from 33% in 1841 to 80% in 2011; the service sector dominates London’s economy (91%); the UK’s economy is more reliant on the service sector than any other G7 country; and the service sector has driven the economic recovery since the downturn in 2008. However, most service sector categories have comparably low labour productivity, and therefore, this is one of the reasons for the UK’s comparatively low overall labour productivity.

As an example of how the dynamics of the UK’s economy could have impacted on its labour productivity value, based on pre-Covid-19 pandemic projections, it is informative to look at pubs and coffee shops, which will come under the category of ‘Food And Beverage Service Activities’; which had an output per hour value of £17.60 for Q4 2018 (half of the median value) and was ranked 60 out of the 67 categories. ONS (2018c) data confirms the large fall in the number of pubs, from around 50,000 pubs in 2008 to around 39,000 pubs in 2018. However, in 2008, UK pubs had a median number of five employees, but by 2018 (partly due to the closure of many smaller pubs), this had increased to eight employees. Therefore, despite the fall in pub numbers, the number of employees in pubs (2015–2018) has stayed around 450,000, higher than at any time since 2002. Over a similar period (2007–2018), the number of UK coffee shops has increased from 10,000 to 24,000, with over three new coffee shops opening every day; a trend that could mean that the number of UK coffee shops overtakes the number of pubs by 2030 (Poulter 2018). This is why Tenreyo’s comment earlier in the paper is misleading: the UK economy is expanding and employment increasing in part because of the continuing trends in ‘Food And Beverage Service Activities’; but because it is a low labour productivity sector, such expansion will drag down the overall value for the UK. One absurd way to increase the UK’s labour productivity would be to close all pubs and coffee shops and make all of the staff unemployed—because this would remove a huge low-productivity component of the UK economy and because the labour productivity index does not allow for the unemployed! Of course, this would be ridiculous, but it serves to highlight a key limitation of the labour productivity index.

By way of comparison, it is interesting to note that Europe’s biggest economy, Germany, promised to phase out coal production by 2038 to meet its climate change commitments. Yet, it has controversially begun expanding one of its biggest open-cast lignite mines, at Hambach, which is 48 square kilometres in size (Oltermann 2019; Thomson 2019). The labour productivity of this development will be very high as it will only involve around 1300 employees because of its using the largest automated machinery in the world (RWE 2020). This serves to highlight how misleading crude international comparisons can be using the unweighted labour productivity index; it is arguably down to luck that such a large, rich mineral deposit so close to the surface happened to be in Germany.

The countries with the highest productivity referred to above (Ireland, Luxembourg and Norway) all have economies skewed towards sectors that have high productivity. This too serves to illustrate the need for any international comparisons of labour productivity to involve standardisation. In another field such as epidemiology, it is possible for one population to have a higher death rate for each age–sex group compared to another population, but have a lower overall death rate, because the age-sex structures of the two populations are very different. Hence, international comparisons utilise standardised mortality (ONS 2018d). There is no reason in theory why this could not equally apply to the labour productivity index, because labour productivity varies between different economic sectors and the balance of economic sectors in different countries will also vary. A country’s standardised productivity rate would not alter as some sectors expand and others contract, as would be likely to happen with the existing labour productivity index, if the sector-specific productivity values remained constant. Of course, this would require labour productivity rates to be calculated for each set sector in each country and then applied to an internationally agreed mix of economic sectors. But until such a development is made, it is inappropriate for the media to wheel out ‘experts’ every time labour productivity figures are published, who then berate UK industry for poor performance with comments along the lines of that made by Tenreyo above. If the uncertain impact of Brexit on the UK’s labour productivity (Bloom et al. 2019) can be put to one side, then the authors suspect that any differentials between the UK and its competitors would reduce, if not disappear in some cases, if standardisation were to take place.

### Limitations

The prime purpose of the research was to determine whether the SLPI represented a valid index which was sufficiently different from the GDPHRWKD to add complementary value. Accordingly, SLPI analyses presented deal with a basic model, from which further research can explore greater sophistication. They necessarily made certain pragmatic assumptions, given that some potentially relevant data were not ready to hand and might not be available for a large number of countries (if indeed it is available in published form at all). One limitation was that a single value of α was assumed in each set of results, when the value will undoubtedly vary between countries and be influenced by whether an economy is expanding or contracting, given the productivity of new employees. Another limitation, highlighted from the outset, was the assumption that the THW of existing workers and newly employed workers are the same. How these limitations might be addressed by further research is discussed below. None of these limitations detracts from the principle that the model presents a useful and complementary measure which addresses the fact that GDPHRWKD does not allow for unemployment.

The assumption that α has a value < 1 is considered to be entirely valid. However, some countries have seen their domestic economies significantly contract as a result of, for example, severe austerity measures; and many will see economic contraction due to the Covid-19 pandemic. As has been described, one by-product of this is that skilled and unskilled workers migrate to other countries to find employment; but this is unlikely to be uniform between countries, for example in the case of the EU. A similar issue relates to cross-border migration, where a person lives in one country and travels to work in another country, such as in the Benelux and neighbouring countries. The impact of both types of migration on the value of α would require a great deal of complex data; and consequently should be the subject of further research.

### Complexity in an uncertain world

As has been highlighted above, there are many complex factors affecting both productivity and unemployment, and the world will be a different place as a result of the Covid-19 pandemic. For example, there is likely to be increased repatriation or re-shoring of manufacturing to the USA and UK (Vanchan et al. 2018), and there is growing concern with the world’s reliance on China’s manufacturing supply chains (Serhan and Gilsinan 2020). Also, there may be changes in migration patterns, and the opportunities that have encouraged migration, which could impact on the size and skill mix of the available workforces, both in countries that have traditionally attracted immigrants and countries from which people have emigrated. Accordingly, the limitations of the labour productivity index may well become exposed. In the absence of the ideally required econometric research to develop new sophisticated economic measurement and productivity statistics, the proposed SLPI can act as a straightforward proxy measure, to complement the labour productivity index. It can be readily utilised for evaluation and monitoring both short- and long-term trends; and identifying outliers which might point to appropriate action and/or research.

### Further research

It is accepted that the SLPI as described can be further developed if relevant data become available. Possibilities relate to the values of α and γ. The value of α reflects the specific labour productivity of the newly employed relative to the existing labour productivity and will be largely influenced by five issues. The first is the sector(s) in which any new jobs occur; for example, if jobs are being lost in high-productivity sectors and replaced by jobs in low-productivity sectors, then the balance will shift accordingly. Second, as stated above, it is likely that there should be values specific to each individual country. Third, it will take some time before an average new employee will achieve optimal productivity; some estimates suggest that it takes 1–2 years (Oakes 2012), and of course, this may be influenced by whether the new employee is full or part-time. Fourth, the impact of significant migration between countries for both skilled and unskilled workers is unlikely to be uniform, as mentioned above. The final issue is whether an economy is in the process of expanding or contracting, as this will impact on the level of new employees. Nevertheless, it would be desirable to undertake research to establish a specific range of values for α that appropriately reflects ‘real-life’, given that this research had to assume a full range from 0.1 to 0.9 in the absence of relevant data. Such a range would then support sensitivity analyses.UK employment statistics are compiled for the Labour Force Survey (ONS 2019c) and this topic might potentially be investigated by microdata analysis of its data.

With regards to γ, it was necessary to assume that the mean THW of all persons engaged in production was the same for both existing and newly employed workers, irrespective of sector or country, because of an absence of relevant data. Whilst being pragmatic, it is unlikely that this will universally be the case, particularly with developments in the ‘gig economy’ and zero-hour contracts. For example, workers on zero-hour contracts accounted for over a quarter (26.8%) of overall employment growth in the UK over the period 2012–2017, having more than tripled from 252,000 to 901,000 workers [It is noted that increased recognition and awareness of these types of contracts led to a large increase between 2012 and 2013, thereby complicating comparisons with 2012 and earlier years (ONS 2014).] Overall, it has been estimated that the UK ‘gig economy’ employs more than 2.8 million workers (Sharma 2018). Therefore, this is a topic that would benefit from greater investigation. Again, Labour Force Survey data might enable related analyses.

The need for a standardised labour productivity index to allow for variations in productivity between different economic sectors and the balance of economic sectors in different countries has been highlighted. Ultimately, this would need to incorporate an internationally agreed mix of economic sectors.

Any similar standardisation relating to the SLPI would be more complex, particularly with regard to setting a value, or values for α. There would also need to be a view taken about how to (consistently) attribute the unemployed to each sector given that some people will move between jobs in different sectors either by choice or because of circumstances, e.g., the demise of one particular sector in a given geographical region will undoubtedly force some people to work in another sector if they do not wish to move away from that region. Such research would only be undertaken following on from the acceptance of the SLPI as a useful complementary measure to accompany labour productivity and the production of standardised labour productivity rates.

## Conclusions

The results show that there is no correlation between countries’ labour productivity and unemployment rates, and that the SLPI provides markedly different patterns over time to the labour productivity index for countries with high(er) unemployment. The Covid-19 pandemic will have a major impact on economies across the world, with reductions in GDP and the speed and degree of recovery inevitably varying. It is projected that there will be associated large increases in unemployment, with the greatest impact likely to be in lower skilled and lower productivity sectors, such as tourism and retail; potentially having the counter-intuitive effect of the labour productivity index actually increasing for some countries, because it only measures the productivity of those in work. The recent trends in international migration (of all types) could well serve to exacerbate the numbers and skill mix of the unemployed. In the circumstances, a wider perspective of productivity is necessary; and levels of unemployment should be taken into account when considering countries’ productivity to gain a broader appreciation of the dynamics of their economies.

The relationship between employment and productivity is complex, and the SLPI represents a practicable high-level proxy measure aimed at countries’ whole workforces and economies. It can be utilised quickly in these unprecedented times to complement the labour productivity index when considering international comparisons of productivity; arguably providing a better measure of countries’ overall economic and social health. It uses data routinely collected and already available, which would be supplemented by assumptions/predictions about values of α and β to model differing potential scenarios. Sensitivity analyses which vary these assumptions can sit alongside GDP and labour productivity index predictions.

The results also showed that the range of existing values for the labour productivity index is so wide that the SLPI does not affect relative rankings of countries, but it is considered that this was more due to vagaries of the labour productivity index. One of these is that in its crude form, the labour productivity index does not allow for countries’ varying balances between economic sectors. Therefore, an internationally agreed standardised labour productivity index should be introduced; the authors anticipate that this would serve to narrow the range of values.

The proposed SLPI in this paper is in effect a basic model that is capable of further development in sophistication with access to more detailed data that were not available to the authors. It is concluded that the SLPI contributes positively to the debate about the labour productivity index and addresses some of its acknowledged limitations.

## Data Availability

Not applicable.
